# The Belgian Refugees at Alexandra Palace

**Published:** 1914-09-26

**Authors:** 


### THE BELGIAN REFUGEES AT ALEXANDRA PALACE.

### Dr. H. Cuff, Medical Superintendent, on Emergency Administration

The newspapers, the squads of recruits passing through the streets, and the unusual frequency of uniforms in Tubes and motor-omnibuses, give to the man in the street the romantic side of war; but if men, and particularly hospital men, wish to see something of its realism, to grasp what administrative problems it instantaneously creates, there is a better way even than talking to the wounded in their wards, and that is to see the refugees from Belgium now congregated at the Alexandra Palace. Wounds may be heroic, but the homeless Flemish peasants and Walloons now brought over have nothing heroic about them. They are simply penniless men, women, and children seeking refuge from a devastated country in a land of whose language and people they are totally ignorant, and are consequently utterly dependent for their food, shelter, and even clothing on the generous hospitality of the strangers to whom they have come in such numbers. These numbers have created institutional problems quite as acute as those caused by the sudden influx of wounded sailors and soldiers into our hospital wards, and, in a sense, more remarkable, since they were quite unforeseen and unprepared for. The centre on which has been cast the strain of dealing with them is, of all places, the Alexandra Palace—the chief emergency "institution" in the country. The story of its conversion in three days from a people's palace into a vast institution with between one thousand and fifteen hundred inmates constantly coming and going is an event in institutional history, which The Hospital's Special Commissioner was fortunate enough to hear described by Dr. Herbert Cuff, Principal Medical Officer of the Metropolitan Asylums Board, who is now acting as medical superintendent in supreme charge of the refugees at the Alexandra Palace.

### How the Work Began.

"As soon as the invasion of Belgium," said Dr. Cuff, "made it necessary for the innumerable refugees from devastated villages and towns to be provided for in this country, I was asked by the Local Government Board to report on the suitability of the Alexandra Palace, Hornsey, as a centre for receiving them on their arrival. As you know, the Palace stands on one of the highest spots in London, and commands a wonderful view over the Metropolis, with the racecourse at its foot, the green slopes just below the actual building, and roofs and spires as far as one can see. If there is fresh air and plenty of space anywhere, it is here. The site is really magnificent for any architectural purpose, but when I came to examine the institutional possibilities of the building, which is a sort of Crystal Palace and museum in one, I saw that there was likely to be one difficulty, that of the heating. The huge size of the galleries provides ample space for any purpose, but with the inner court and glass halls the place is apt to be draughty. Had the time been April, I should have said that no better place could be found. But it will soon be cold, and I shall be obliged to put gas-stoves in some of the halls and galleries, and a large stock of warm clothing is accumulating."

Walking with Dr. Cuff down the eastern aisle of the building, as he said these words, a curious scene surrounded us. Men, women, and children, mingled higgledy-piggledy, were on all sides, in large and small groups, sitting, standing, talking, eating, while the sound of children playing in swings and on a hand-driven roundabout close at hand gave the air of a Bank Holiday crowd, except for the absence of colour in hats and dresses, and the absence of singing and laughter.

"How many have you here?" I asked.

"Four hundred and fifty arrived on Monday. They came mostly at ten or eleven at night, exhausted after their long journey, hungry, tired, and some still hardly recovered from sea-sickness. On Tuesday 182 came, on Wednesday we had 780, on Thursday 170, and to-day (September 18) two or three hundred more are expected. You see the whole place is absolutely an improvisation. We all, my staff and the refugees, only arrived three days ago. The point of the procedure is this. When the refugees arrive in London the more well-to-do are met at the stations by representatives of the War Refugees' Committee and the League of Catholic Women, who are armed with lists of cheap hotels, apartments, and addresses of private offers of accommodation. But the poorest class, the Belgian peasantry, are brought here direct, where they remain sometimes a day or two, sometimes longer, till they can be selected by the allocation committees. Parties are despatched from here daily to all parts of the country, and at all hours of the day."

At this point Dr. Cuff, who has to be everywhere at once and decide the most diverse points at a moment's notice, was, naturally not for the first or last time, interrupted for a moment. No sooner was I left standing than a small crowd of refugees surrounded me, all holding out for my inspection the luggage labels which were attached to their dresses or coats. On scrutinising these I read a name, either Dutch or Belgian, and the name of a place—Shrewsbury, Westbury, Hampstead, were all examples. I could not understand their language, which sounded like Flemish, and was in despair until a woman who could speak French joined the circle. Then it became clear that one question was agitating the throng: Could I tell them how far each place was to which they were going? Shrewsbury? "Two hundred kilometres," I replied, unhesitatingly, knowing that a definite answer was better than none at all. Westbury? "One hundred kilometres." These guesses I have not dared to verify. But the look of dismay with which the larger figure was heard, and the pleasure which greeted the remark that Hampstead was close by, showed that most of the refugees had had enough of travel. The husband of the Frenchwoman was a prisoner in Germany, she said. Dr. Cuff's return prevented me from disseminating any further inaccuracies, and the complicated questions involved in standing *in loco patriæ* to these unfortunates became every moment more clear.

### Emergency Catering.

"How are they all catered for?"

"The central hall is used for meals, as you see, and the catering is done by the catering department of the Metropolitan Asylums Board. To the Board's extraordinarily varied work this new undertaking has been added, and is it not natural that we think that some better name than the old one is needed to express how far our work has outgrown that laid down in 1867, when the so-called Asylums Board was constituted? Our own department does all the catering. I have to assist me Mr. Gibbs, the steward from Tooting Bec Asylum, and Mr. Wells, steward at the North-Eastern Fever Hospital, Tottenham.

"On their arrival the refugees are brought straight to the central hall, where bread and soup are immediately served. Food is their first necessity, but, as some are still hardly recovered from sea-sickness, we have tried more than one sort of meal. Cake and coffee was yesterday's experiment, but I am not sure if this is really preferred. As a matter of fact, one of the first things done was to consult the Belgian Legation as to the most suitable menus. You see, they are all peasants, and I wanted to give them, so far as possible, what they were accustomed to at home. For from every point of view we keep in the foreground of our consciousness the fact that, however poor these refugees may be—and it is the poorest mostly that come here—they are not to be treated as casuals, but as our guests. The Legation informed me that they were unaccustomed to meat, and we procured a Belgian cook, not a refugee, to prepare the meals. But I have come to the conclusion that they do want meat, and so their dietary consists of a breakfast of coffee, bread and margarine; dinner, a basin of stew which contains meat, with as much bread as they like; and supper at night of bread, margarine, and coffee, or, in the case of children, of hot milk."

"But what staff have you for the service and so on?"

"The Palace staff has been kept on, and my right hand here is Mr. Wheatley, the resident general manager. Without his help I never could have coped with the difficulties at all. There is nothing he cannot do, from building baths and latrines to providing amusements for the children. From the refugees I have picked a number of men and women, the former of whom help to wait at table and do the washing-up, while the women assist in scrubbing the floors of the dormitories and dusting the rooms. I may say at once that they seem only too delighted to have something of this kind to do, and consent without the smallest objection. Otherwise, as you see, instead of going out into the grounds and gardens, they huddle together indoors in the galleries, talking or doing nothing in particular. I am, however, arranging for one of the rooms to be filled with tubs, where the women can wash their clothes and their babies."

The Sleeping Arrangements.

"I see some sleep in the central hall by the dining-room."

"These beds have not been used as yet, but, as in the other sleeping rooms, you will see that the refugees are provided with a straw mattress laid on the floor, a straw pillow and a pillow-slip, and a couple of blankets apiece. The women and children are together on one side of the building and the men on the other. The mattresses are placed in rows with chairs back to back between each. There are at present no sheets, and, in fact, the question of sheets has been rather a problem. Mr. Jury, the head of the sanitary department of the London County Council, has come to advise me on the subjects of bedding and sanitation. To provide sheets for all these mattresses will be a costly procedure, but, speaking from his wide experience of common lodging-houses, Mr. Jury says that the value of sheets lies in the ease with which vermin are detected in them, and also prevented from entering the blankets. All the refugees require cleansing on their arrival, and they are being taken each day in London General motor omnibuses to the Wood Green and Hornsey baths until the baths which are now being built in the old monkey-house are complete. This bathroom will take the form of galvanised iron baths placed in cubicles formed by cretonne and wooden laths. Before coming here I was in charge of some Russian Jews, refugees, at Poland Street, Westminster, and by a piece of good fortune their quarters communicated directly with the Westminster Baths. But here until our own are built we have to drive to the baths at Wood Green and Hornsey. The cleansing of their clothes is also a problem, but we have only had time so far to convert the choir and orchestra seats by the organ in the central hall into an impromptu cloak room. Each refugee brings his or her spare bundle—all they have been able to carry away—to the orchestra, where it is placed on one of the numbered orchestral seats. The depositor receives a counterfoil similarly numbered, and so without registering names of any kind their parcels are obtainable on demand. You can see the parcels from here grouped on the rising tiers of the orchestra."

Sanitary and Allied Problems.

"Have you had much difficulty with the sanitary arrangements?"

"These are rapidly being brought up to our present requirements. Of course, a number of lavatories are in existence, as the Palace in an ordinary way is open all the year round. But this is the first time that the Palace has been subjected to a resident crowd. Eighty latrines are being built, and we are able to connect them with the main drains, so that this problem has not been very difficult. The sort of thing that has been a difficulty has been in the kitchen, which possessed no institutional apparatus for preparing the very large supplies of tea, coffee, or soup which are required for the large numbers who arrive. Again, the ventilation of these very lofty rooms is far from perfect. I am having the semicircular tops of the high windows all made to open to-day. One of my difficulties is the huge size of the place, which causes one to do a tremendous lot of walking."

Better-class Accommodation.

"Among the crowd of peasants there are a few who are more well-to-do. These are quartered in what are in ordinary times the rooms of the Welcome Club, a body which leases its premises from the trustees of the Palace. Here they have a dining-room, which at present has also to serve as a dormitory at night, and a writing-room, and, in short, more quiet and more privacy. The nurses under Miss Cockerell, the able matron of Marylebone Infirmary, are also quartered in this part of the building. I must now conclude our round with the sick ward."

The Sick Ward.

"I have set apart this one room, which, as you see, is large enough to contain perhaps twenty beds, though, being a square room, these have to be placed in rows. The wooden floors are being scrubbed, and there are only to-day some half a dozen occupants. Any really serious case would be sent to Edmonton Infirmary, where already one or two women in an advanced stage of pregnancy have been removed."

"You have no idea how long the work here will be required?"

"None whatever."

With these words Dr. Cuff gave himself up to other claimants on his time, and as I walked down the green hill from the crowded galleries of the Palace, I passed a squad of English recruits drilling in the grounds. It was the other side of the work I had just left.

